# 127. Evaluation of Real-Time Verigene Blood Culture Alerts on Time to Optimal Antibiotic Therapy

**DOI:** 10.1093/ofid/ofad500.200

**Published:** 2023-11-27

**Authors:** David T Adams, Satwinder S Kaur

**Affiliations:** Texas Health Harris Methodist Hospital Fort Worth, Fort Worth, Texas; Texas Health Harris Methodist Hospital Fort Worth , Dallas, Texas

## Abstract

**Background:**

Verigene blood culture (VBC) results can help facilitate reductions in time to optimal antimicrobial therapy (TTOT), potentially leading to improved clinical outcomes. We sought to evaluate if TTOT could be further reduced by implementing automated real-time VBC alerts delivered to the antimicrobial stewardship (AMS) team.

**Methods:**

A retrospective observational cohort study of adult patients with bacteremia and a VBC result from May to September 2021 comparing outcomes when alerts resulted while AMS team members were working compared to not working. AMS team members review all positive blood culture alerts and make recommendations to the treating physicians based on results and current antimicrobial regimens. Excluding patients with a VBC result of *Staphylococcus* species, *Streptococcus* species, *S. epidermidis*, or no target detected, patients with an opportunity for change in their empiric antimicrobial regimen based on the VBC result were included.

**Results:**

In total, 207 patients met inclusion. The most frequent organisms encountered with an opportunity for intervention were *E. coli* (n=92) and *S. aureus* (n=49). The most common source was urinary (37.2%). Mean TTOT was shorter in the VBC+AMS group (41.4 hours) compared to the VBC only group (53.1 hours) regardless of whether the AMS team recommendation was accepted, *P* = .035. This difference was unsurprisingly larger when evaluating only those in the VBC+AMS group where the recommendation was accepted (18.9 hours) compared to all other patients (54.6 hours), *P* = < .0001. All-cause in-hospital mortality was significantly lower in the VBC+AMS group (12.3%) compared to the VBC only group (26.9%), *P* = .02. Similar rates of change to optimal therapy at any point during the admission (91%) were observed in the VBC only and VBC+AMS groups.

**Conclusion:**

The implementation of a real-time alert system to communicate VBC results to the AMS team resulted in a significant reduction in TTOT and in-hospital mortality when alerts occurred while AMS team members were working. Given the high antibiotic optimization rates in both groups but a difference in mortality, further studies are needed to identify if there is an association between TTOT and improved clinical outcomes beyond the benefits of timely effective antimicrobial therapy.

**Disclosures:**

**All Authors**: No reported disclosures

